# First Viruses Infecting the Marine Diatom *Guinardia delicatula*

**DOI:** 10.3389/fmicb.2018.03235

**Published:** 2019-01-09

**Authors:** Laure Arsenieff, Nathalie Simon, Fabienne Rigaut-Jalabert, Florence Le Gall, Samuel Chaffron, Erwan Corre, Emmanuelle Com, Estelle Bigeard, Anne-Claire Baudoux

**Affiliations:** ^1^Sorbonne Université, CNRS UMR 7144, Diversity and Interactions in Oceanic Plankton - Station Biologique de Roscoff, Roscoff, France; ^2^Sorbonne Université, CNRS Fédération de Recherche FR2424 - Station Biologique de Roscoff, Roscoff, France; ^3^Laboratoire des Sciences du Numérique de Nantes (LS2N), CNRS UMR 6004 – Université de Nantes, Nantes, France; ^4^Univ Rennes, Inserm, EHESP, Irset (Institut de recherche en santé, environnement et travail) - UMR_S 1085, Rennes, France; ^5^Protim, Univ Rennes, Rennes, France

**Keywords:** single-stranded RNA viruses, diatoms, genomics, host-virus dynamics, Western English Channel

## Abstract

The marine diatom *Guinardia delicatula* is a cosmopolitan species that dominates seasonal blooms in the English Channel and the North Sea. Several eukaryotic parasites are known to induce the mortality of this species. Here, we report the isolation and characterization of the first viruses that infect *G*. *delicatula*. Viruses were isolated from the Western English Channel (SOMLIT-Astan station) during the late summer bloom decline of *G*. *delicatula*. A combination of laboratory approaches revealed that these lytic viruses (GdelRNAV) are small tailless particles of 35–38 nm in diameter that replicate in the host cytoplasm where both unordered particles and crystalline arrays are formed. GdelRNAV display a linear single-stranded RNA genome of ~9 kb, including two open reading frames encoding for replication and structural polyproteins. Phylogenetic relationships based on the RNA-dependent-RNA-polymerase gene marker showed that GdelRNAV are new members of the *Bacillarnavirus*, a monophyletic genus belonging to the order *Picornavirales*. GdelRNAV are specific to several strains of *G*. *delicatula*. They were rapidly and largely produced (<12 h, 9.34 × 10^4^ virions per host cell). Our analysis points out the host's variable viral susceptibilities during the early exponential growth phase. Interestingly, we consistently failed to isolate viruses during spring and early summer while *G*. *delicatula* developed important blooms. While our study suggests that viruses do contribute to the decline of *G*. *delicatula*'s late summer bloom, they may not be the primary mortality agents during the remaining blooms at SOMLIT-Astan. Future studies should focus on the relative contribution of the viral and eukaryotic pathogens to the control of *Guinardia*'s blooms to understand the fate of these prominent organisms in marine systems.

## Introduction

Diatoms are a major component of phytoplankton communities. They have a worldwide distribution (Mann and Droop, 1996; Malviya et al., 2016), occurring in freshwaters and marine habitats from the poles to the tropics (Takano, 1981; Kellogg and Kellogg, 1996; Sarno et al., 2005; Hernández-Becerril et al., 2010; Balzano et al., 2017). They are responsible for 35–75% of the marine primary production in the oceans (Nelson et al., 1995) and they play a fundamental role in the transfer of carbon to consumers (Armbrust, 2009). They are also important drivers in the ocean's export production due to their high sinking rate, thus playing a key-role in the functioning of the biological carbon pump (Falkowski et al., 1998; Smetacek, 1999). In nutrient rich coastal ecosystems, diatoms produce recurrent seasonal successions of species and blooms (Assmy and Smetacek, 2009; Sommer et al., 2012). The marine diatom genus *Guinardia* is described as a considerable contributor to micro-phytoplankton assemblages along the Atlantic coasts, in the English Channel (Grall, 1972; Gómez and Souissi, 2007; Guilloux et al., 2013), North Sea (Wiltshire et al., 2010) and western Irish Sea (Gowen et al., 1999). Especially, in the German Bight at Helgoland Roads time series, the bloom-forming species *Guinardia delicatula* is one of the most abundant diatom species, with highest abundances in early summer and autumn. However, a trend toward an earlier and wider period of development in response to environmental variables has been detected (Wiltshire et al., 2010; Schlüter et al., 2012). In the Western English Channel (WEC), *G*. *delicatula* dominates the seasonal cycle production, where its spring-summer development occurs commonly from May to August/September (Grall, 1972; Guilloux et al., 2013; Simon et al., personal communication).

Decades of research have emphasized the decisive role of physical factors (e.g., light, turbulence, and sedimentation), nutrient limitations and predation by zooplankton in pacing the seasonal development of marine diatoms (Smetacek, 1985; Sarthou et al., 2005; Schlüter et al., 2012; Sommer et al., 2012). Parasites have also been identified as potential primary agents that could shape diatom population dynamics (Tillmann et al., 1999; Gleason et al., 2015; Scholz et al., 2015). In the literature, several eukaryotic parasites of the genera *Pirsonia* (Kühn et al., 1996), *Cryothecomonas* (Drebes et al., 1996) and *Rhizamoeba* (Kühn, 1996) were described associated with *G*. *delicatula*. More recently, viruses have been identified as mortality agents involved in the control of diatoms dynamics. Up to date, about 20 diatom viruses have been described. They are separated into two groups: the single-stranded RNA (ssRNA) viruses (Shirai et al., 2008; Tomaru et al., 2009; Kimura and Tomaru, 2015) and the single-stranded DNA (ssDNA) viruses (Toyoda et al., 2012; Tomaru et al., 2013b; Kimura and Tomaru, 2015). Viruses of diatoms are also highly specific to their hosts, with species-specificity or even strain-specificity (Nagasaki et al., 2004; Tomaru et al., 2008; Toyoda et al., 2012). Different viruses infecting the diatom *Chaetoceros tenuissimus* can display variable environmental optima suggesting a niche partitioning in the nature (Kimura and Tomaru, 2017). As a consequence, viruses may control diatoms over a broad environmental range and play a key role in species or infra-specific groups successions. Nevertheless, more isolations and characterizations are needed to better understand the role of viruses in the regulation of host populations. To our knowledge, no virus is known to infect *G*. *delicatula*.

In this study, we isolated four ssRNA viruses causing lysis of *G. delicatula* from the long-term monitoring SOMLIT-Astan station located off Roscoff (Western English Channel, WEC). The host range, morphological features, lytic cycle, genome structure and phylogenetic position of the representative GdelRNAV-01 (*G. delicatula* ssRNA virus 01) were fully described. These viruses are new members of the *Bacillarnavirus* genus within the *Picornavirales* and share common features with other viruses infecting diatoms. Due to the ecological importance of its host, this discovery raises new questions about the contribution of viruses and other parasites to the interaction network associated with *G*. *delicatula* in the WEC.

## Experimental Procedures

### Growth Conditions of Algal Cultures

The marine diatom *G. delicatula* RCC3083 has been used in this study for the isolation of viruses. This xenic clonal strain was provided by the Roscoff Culture Collection (RCC, http://roscoff-culture-collection.org/) and was isolated the 19th September 2012 from surface water at the Roscoff Estacade station in the Western English Channel (48:43:56 N, 3:58:58 W). *G*. *delicatula* RCC3083 was maintained in sterile condition in K+Si medium (Keller et al., 1987) at 18°C, under a 12:12 h light:dark cycle of 100 μmol photons·m^−2^·s^−1^ provided by a white fluorescent light (Philips Master TL_D 18W/865). These culture conditions were used for all the following experimentations.

### Genetic Variations Among *G*. *delicatula* Strains

Intraspecific variability within *G. delicatula* was examined in the SSU-18S, ITS and partial LSU-28S rDNA genes markers. The primers used were 63F (ACGCTTGTCTCAAAGATTA) and 1818R (ACGGAAACCTTGTTACGA) (Lepere et al., 2011) for the 18S, 329F (GTGAACCTGCRGAAGGATCA) (Guillou et al., 2004) and D1R-R (TATGCTTAAATTCAGCGGGT) (Lenaers et al., 1989) for the ITS, and D1R-F (ACCCGCTGAATTTAAGCATA) (Lenaers et al., 1989) and D3Ca (ACGAACGATTTGCACGTCAG) (Orsini et al., 2002) for the partial 28S D1-D3 region. Briefly, aliquots (2.25 μL) of *G*. *delicatula* cultures (Table 1) were submitted to 95°C for 5 min. The reaction mixture (30 μL final volume) was then added: Phusion Master Mix (1× final concentration, Thermo Scientific), 3% DMSO and 0.25 μM of each primer. PCR amplifications were performed with the following conditions: an initial incubation step at 95°C for 5 min, followed by 35 or 40 cycles (respectively for 18S−28S and ITS genes) of denaturation at 95°C for 1 min, annealing step for 30 s at 55, 52, and 57° for the amplifications of the SSU, ITS, and LSU, respectively, and extension at 72°C for 1 min 30. The cycles were followed by a final extension step at 72°C for 10 min. PCR products were sent for Sanger sequencing to GATC Biotech (https://www.gatc-biotech.com/en/index.html, Constance, Germany). Sequences were analyzed and aligned using Geneious 9.1.3.

**Table 1:** Host range of GdelRNAV viral strains: lytic activity recorded within the species *Guinardia delicatula* and for other phytoplankton species.

**Phylum**	**Class**	**Species**	**Strain code**	**Origin of isolation**	**Date of isolation**	**Lysis by GdelRNAV-01**	**Lysis by GdelRNAV-02**	**Lysis by GdelRNAV-03**	**Lysis by GdelRNAV-04**
Bacillariophyta	Coscinodiscophyceae	*Guinardia delicatula*	**RCC3083**	Roscoff Estacade, EC	19/09/2012	++	++	++	++
			RCC4834	Penzé estuary, EC	24/05/2015	–	–	–	–
			RCC5777	Roscoff-Astan, EC	21/10/2015	–	–	–	–
			RCC5778	Roscoff-Astan, EC	21/10/2015	–	–	–	–
			RCC5779	Roscoff-Astan, EC	21/10/2015	–	–	–	–
			RCC5780	Roscoff-Astan, EC	21/10/2015	–	–	–	–
			RCC5781	Roscoff-Astan, EC	21/10/2015	–	–	–	–
			RCC5782	Roscoff-Astan, EC	04/11/2015	+	+	+	+
			RCC5783	Roscoff-Astan, EC	29/04/2016	+	+	++	++
			RCC5784	Roscoff-Astan, EC	13/05/2016	+	+	+	+
			RCC5785	Roscoff-Astan, EC	13/05/2016	+	–	–	–
			RCC5787	Roscoff-Astan, EC	23/09/2016	++	++	++	++
			RCC5788	Roscoff-Astan, EC	24/10/2016	–	–	–	–
			RCC5789	Roscoff-Astan, EC	19/05/2017	–	–	–	–
			RCC5790	Roscoff-Astan, EC	02/06/2017	–	–	–	–
		*Guinardia flaccida*	RCC3093	Roscoff-Astan, EC	19/09/2012	–	–	–	–
		*Guinardia striata*	RCC5792	Roscoff-Astan, EC	09/09/2016	–	–	–	–
			RCC5793	Roscoff-Astan, EC	23/09/2016	–	–	–	–
		*Rhizosolenia* sp.	RA170220	Roscoff-Astan, EC	20/02/2017	–	–	–	–
	Mediophyceae	*Thalassiosira punctigera*	RCC4667	Roscoff-Astan, EC	21/10/2015	–	–	–	–
		*Thalassiosira curviseriata*	RCC5154	Roscoff-Astan, EC	26/05/2015	–	–	–	–
		*Thalassiosira* sp.	RCC4659	Roscoff-Astan, EC	26/05/2015	–	–	–	–
		*Minidiscus variabilis*	RCC4657	Roscoff-Astan, EC	26/05/2015	–	–	–	–
		*Minidiscus comicus*	RCC4660	Roscoff-Astan, EC	26/05/2015	–	–	–	–
		*Detonula pumila*	RCC5794	Roscoff-Astan, EC	13/07/2016	–	–	–	–
		*Chaetoceros peruvianus*	RCC2023	Roscoff-Astan, EC	01/09/2010	–	–	–	–
	Bacillariophyceae	*Nitzschia* sp.	RCC80	Roscoff Estacade, EC	01/06/1997	–	–	–	–
		*Pseudo-Nitzschia* sp.	RCC3101	Bay of Concarneau	12/06/2012	–	–	–	–
Miozoa	Dinophyceae	*Prorocentrum micans*	RCC3046	Penzé estuary, EC	01/01/2006	–	–	–	–
Haptophyta	Prymnesiophyceae	*Phaeocystis* sp.	RCC1000	MAR4, Marquesas islands	29/10/2004	–	–	–	–

*In bold, host strain used for the isolation of GdelRNAV strains. EC, English Channel; RCC, Roscoff culture collection; ++, complete lysis; +, partial lysis (healthy host cells still present after 14 days of inoculation); –, no lysis*.

### Temporal Dynamics of *G. delicatula*

Seasonal variations in abundance of the diatom *G*. *delicatula* at the long-term monitoring station SOMLIT-Astan off Roscoff (48:46:18 N, 3:58:6 W) were obtained from microscopic counts data (RESOMAR-Pelagos database, http://abims.sb-roscoff.fr/pelagos). Briefly, surface seawater (1 meter depth) collected bi-monthly was preserved in acidic Lugol's iodine solution. After sedimentation in Utermöhl chambers, cell counts and identifications were performed under an inverted light microscope (Guilloux et al., 2013).

### Isolation of Viruses

Samplings were conducted every fortnight between October 2015 and October 2016 at SOMLIT-Astan station. This station is representative of the permanently mixed water column of the Western English Channel (Wafar et al., 1983; L'Helguen et al., 1996). Seawater samples of 3L were collected at 1 m depth using a 5 L Niskin bottle. Back in the laboratory, samples were immediately pre-filtered through a 150 μm pore-size nylon filter to remove most of the micro- and mesozooplankton. 250 mL of pre-filtered samples were enriched with F/2 medium (10% v/v) and 5 mL of culture of *G*. *delicatula* RCC3083. After 2 weeks of incubation, the enriched samples were successively filtered through a GF/F filter (Whatman) and 0.22 μm PES filter (Whatman) to isolate the viral community.

Aliquot (0.5 mL) of the 0.22 μm-filtered samples were inoculated into 1.5 mL exponentially growing host culture in 24-multiwell plates under the host culture conditions as described above. Untreated host cultures served as controls.

Cultures were inspected by light microscopy 2 weeks after inoculation. If algal lysis was observed, 3 extinction dilution cycles were carried out to clone the pathogens (Suttle, 1993). Briefly, 100 μL aliquots of the lysates were serially diluted in 10-fold increment in 900 μL of exponentially growing culture of *G*. *delicatula* RCC3083 (900 μL). Lysates in the last dilution before extinction were transferred to another exponentially growing culture of *G*. *delicatula* RCC3083 and a new filtration on 0.22 μm was repeated to verify the transferability.

From this procedure, 4 clonal viral isolates lytic to *G*. *delicatula* strain RCC3083 have been obtained: GdelRNAV-01 (RCC5809), GdelRNAV-02 (RCC5810), GdelRNAV-03 (RCC5811), and GdelRNAV-04 (RCC5812). They were isolated from natural samples collected respectively on the 23th September, 26th August, 09th September, and 24th October 2016. They were maintained in culture by bimonthly transfers in *G*. *delicatula* RCC3083 under the host culture conditions described previously.

### Host Ranges

To study the host ranges of GdelRNAV-01, GdelRNAV-02, GdelRNAV-03, and GdelRNAV-04, the viral suspensions were filtered on 0.45 μm PES filter (Whatman) and were added to 29 exponentially growing phytoplankton cultures (10% vol/vol), including 27 diatom cultures (Table 1). Untreated phytoplankton cultures served as controls. The experiment has been carried out in triplicate.

Algal growth and lysis were monitored after 7 and 14 days post-inoculation (dpi) under light microscopy. After 2 weeks incubation, phytoplanktonic cultures where no lysis was detected were not considered as susceptible hosts for these clonal viruses.

### Transmission Electronic Microscopy (TEM)

To inspect the replication site of GdelRNAV-01, an exponential culture of *G*. *delicatula* RCC3083 host strain was inoculated with a fresh 0.45 μm filtered virus lysate (5% vol/vol). Uninfected host served as control. Aliquots of the cell suspensions were sampled every 12 h post-inoculation (hpi), and the algal abundances in the control and infected cultures were monitored by optical microscopy (Sedgewick Rafter, Hausser Scientific, USA). 10 mL of the aliquots were fixed with 1% glutaraldehyde and stored at 4°C until treatment. Pluronic F68 (final concentration 0.01%, Gibco) was added and cells were pelleted by centrifugation. Samples were rinsed twice in K+Si medium and 0.2 M cacodylate buffer (pH = 7.53) containing 2% of NaCl were added. Samples were then fixed with 1% OsO4 for 1 h at 4°C. After three washings with the cacodylate buffer, samples were progressively dehydrated in ethanol series (from 30 to 100%). Samples were embedded in Spurr's epoxy resin (Low viscosity, Electron Microscopy Sciences) and were polymerized over a week-end at 60°C. Thin sections (40–70 nm) were cut using a Leica ultracut UCT microtome and mounted on copper grids. Sections were stained with 0.4% uranyl acetate and viewed with a JEOL-JEM 1400 electron microscope (JEOL Ltd., Tokyo, Japan) operating at 80 kV.

Morphological features of the virions were also determined by TEM. Briefly, a fresh viral lysate of each four viral strains was filtered through 0.22 μm pore size filter and concentrated by centrifugation (Vivaspin 50 kDa, Sartorius). Concentrated viral suspension was negative stained for 40 s using uranyl acetate (2% w/v) on a copper grid. Appropriate controls (filtrates from uninfected hosts) have also been examined by TEM.

### Growth Experiment

In order to study the virus growth kinetics, triplicates of exponentially growing cultures of *G*. *delicatula* RCC3083 were inoculated with a fresh 0.1 μm filtered suspension of GdelRNAV-01 (10% v/v, with a multiplicity of infection of 359.5). An untreated culture of *G*. *delicatula* RCC3083 served as control. Samples were taken every 12 h for 8 days to monitor host and virus parameters. Diatom counts were obtained using a Sedgewick Rafter cell (Hausser Scientific, USA) on an inverted microscope. Epifluorescence microscopy (U-MNB2 filter, Olympus BX51, Tokyo, Japan) was used to monitor morphological changes occurring in chloroplasts (using the fluorescence of Chl *a*) and in PicoGreen stained nuclei (Picogreen, final concentration 1×, Molecular Probes).

Viral titer was measured using the extinction dilution method (Suttle, 1993) and was estimated with the software Most Probable Number (MPN; version 2.0, Avineon, U.S Environmental Protection Agency). This experiment was performed in duplicate.

Viral latent period was calculated as the period of time between the viral inoculation and the first increase in viral titer. Burst size (number of viral progenies produced per one host cell lysed) was estimated from the ratio between the increase in viral titer and the decline in host cell concentration for a given period (from 72 to 96 h in our case), as:

$$\begin{array}{l} BS=\frac{V_{max}-V_{min}}{H_{max}-H_{min}} \end{array}$$

where *V*_max_ and *V*_min_ are the maximal and minimal viral concentrations, respectively, and *H*_max_ and *H*_min_ the maximal and minimal host abundances.

### Virions Thermal Stability

The stability of the virions to temperature was determined by incubating 0.5 mL of a 0.2 μm filtered GdelRNAV-01 suspension at −196, −80, −20, 4, 10, 18, 25, 30, 40, 50, and 60°C. After 24 h, samples were thawed or cooled down for 30 min at room temperature and inoculated with *G*. *delicatula* RCC3083 (10% v/v) in triplicates in 48-multiwell plates. Cultures were inspected by light microscopy at 7 and 14 days dpi to detect lysis.

### Sensitivity to Chloroform

In order to determine whether GdelRNAV-01 is enveloped by a lipid membrane, 10% and 50% (v/v) of chloroform were added to aliquots (1.5 mL) of 0.2 μm filtered lysate. The mixtures were vigorously homogenized by inversion and incubated for 60 min at room temperature. Chloroform was removed by centrifugation, 2,200 × *g* for 20 min at room temperature and the aqueous layers, containing the virions, were transferred to new tubes. Samples were left overnight for evaporation to remove any chloroform contamination. Negative controls of K + Si medium were included. Samples were inoculated with *G*. *delicatula* RCC3083 (10% v/v) in triplicates in 48-multiwell plates. Cultures were inspected by light microscopy at 7 and 14 days dpi.

### Virus Purification

A freshly produced GdelRNAV-01 lysate (500 mL) was filtered through 0.45 and 0.1 μm PES filters to remove cellular debris and bacteria. Polyethylene glycol 6000 (PEG, Sigma Aldrich) was added to the filtrate (final concentration 10% wt/v) and stored at 4°C overnight as described in Tomaru et al. (2004). The mixture was centrifuged at 30,100 × *g*, 4°C, for 2h15 (Avanti J-26XP, Beckman Coulter) and the pellet containing the viruses was washed with 10 mM phosphate buffer (10 mM KH_2_PO_4_ and 10 mM Na_2_HPO_4_, pH 7.2). The suspension was transferred to a Falcon tube (polypropylene) and an equal volume of chloroform was added. The sample was vigorously vortexed and centrifuged at 2,200 × *g*, for 20 min at room temperature. The aqueous layer was recovered and the chloroform procedure was repeated 7 times. After ultracentrifugation (207,870 × *g*, 4 h, 4°C, 70 Ti rotor, Optima XPN-80, Beckman Coulter) of the last aqueous phase, the viral pellet was collected and resuspended in 500 μL of Nuclease-Free Water (Life Technologies). This purified virus sample was used for analysis of nucleic acids, genome sequencing and analyses of structural proteins.

### Viral Nucleic Acids

The nucleic acids (300 μL) of the four viral strain suspensions were extracted using the Kit MasterPure complete DNA and RNA purification (Epicenter) according to the manufacturer's instructions. This extraction was performed on a purified viral suspension for GdelRNAV-01 and on non-purified suspensions of GdelRNA-02, GdelRNA-03 and GdelRNA-04. Around 50 mL of lysates were filtered through 0.45 μm and 0.1 μm PES filters and concentrated by centrifugation (Vivaspin 50 kDa, Sartorius).

To determine the nature of viral nucleic acids, enzymatic digestions of nucleic acids extracts were conducted. Aliquots of 4 μL were digested with DNase I (final concentration 0.05 U·μL^−1^, Epicenter) at 37°C for 1h, with RNase A (final concentration 0.025 μg·μL^−1^, Epicenter) or with S1 nuclease (final concentration 0.03 U·μL^−1^, Promega) that degrades single stranded nucleic acids, for 30 min at room temperature. An untreated aliquot was kept on ice to serve as control. After incubation, samples were resolved on 1.2% agarose gel, stained with ethidium bromide and electrophoresed at 100 V for 50 min. The gel was visualized on Imagequant LAS4000 (GE Healthcare, Waukesha, WI, USA).

The ssRNA viral nucleic acids were converted to cDNA using the SuperScript III Reverse Transcriptase (Invitrogen) with random primers (Hexamers, 250 ng/μL) following the manufacturer's protocol.

### Viral Genome Sequencing and Analyses

The complete viral genome of GdelRNAV-01 was obtained from a 2 × 150 bp paired-end run sequencing on an Illumina NextSeq platform performed by Fasteris (https://www.fasteris.com/dna/, Plan-les-Ouates, Switzerland). A total of 42,872,641 paired reads of 150 nt were quality trimmed using Trimmomatic v. 0.33 with default parameters (Bolger et al., 2014) and normalized using the Diginorm script accessible in the Trinity assembler package (Grabherr et al., 2011). The 443,025 remaining reads were *de novo* assembled into scaffolds with SPAdes version 3.11.0 using a combination of Kmer size 21, 33, 55 and 77 (Bankevich et al., 2012). Scaffolds sequences larger than 8,000 nucleotides were analyzed by megablast against nr database (release February 2018) and blastx against viral section of nr database (release February 2018) leading to the detection of a unique scaffold of 9,233 nucleotides matching viral sequences. Genes prediction was performed using NCBI ORFfinder (https://www.ncbi.nlm.nih.gov/orffinder/) and validated by NCBI SmartBLAST (http://blast.ncbi.nlm.nih.gov/smartblast/).

### Viral Proteins

An aliquot of purified viral suspension (75 μL) was boiled in 4× Laemmli buffer (25 μL) for 5 min and incubated on ice for 30 min. The mixture was then resolved on SDS-PAGE gel (NuPAGE 4–12% Bis-Tris Protein Gel, Life Technologies) using an XCell SureLock Mini-Cell (Invitrogen, Carlsbad, CA, USA) at 200 V for 45 min. After migration, the gel was rinsed 3 times in MilliQ water and stained overnight with ProSieve EX Safe Stain (Lonza Rockland, Inc). The gel was destained in MilliQ water baths and visualized with a white light table. Bands were excised and digested with trypsin for analysis by mass spectrometry using an Orbitrap instrument (LTQ-OrbitrpXL, Thermo Scientific) on Protim platform (https://www.protim.eu/, Rennes, France) as previously described (Lavigne et al., 2012) (see Supplementary Material). The mass spectrometry proteomics data have been deposited to the ProteomeXchange Consortium via the PRIDE (Vizcaíno et al., 2016) partner repository with the dataset identifier PXD010967 (project accession) and 10.6019/PXD010967 (project DOI).

### Phylogenetic Analysis of ssRNA Viruses

In order to determine the taxonomic position of GdelRNAV-01, its closest relatives were searched in NCBI non-redundant database (release 01 February 2018) using the helicase, RdRp and ORF2 amino acid sequences as query with the BLASTP tool (https://blast.ncbi.nlm.nih.gov/Blast.cgi). The phylogenetic position of GdelRNAV-01 was inferred from comparative analyses of amino acid sequences encoding the RNA dependent RNA polymerase (RdRp) domain of the replicase polyprotein. The sequence of the GdelRNAV-01 RdRp domain was retrieved from the whole genome sequence using the Basic Local Alignment Search Tool (BLAST, https://blast.ncbi.nlm.nih.gov/Blast.cgi). RdRp domain sequences of a selection of *Picornavirales* that are representative of different families (International Committee on Taxonomy of Viruses, ICTV, https://talk.ictvonline.org/) were selected. The sequence alignment was generated by the MAFFT version 7 program and the E-INS-I iterative refinement method (https://mafft.cbrc.jp/alignment/server/, Katoh et al., 2017). A phylogenetic tree was constructed by maximum likelihood with PhyML 3.0 (http://www.atgc-montpellier.fr/phyml/, Guindon et al., 2010) with the automatic model selection by SMS (Lefort et al., 2017) and 1,000 bootstrap replicates. MEGA7 (Kumar et al., 2016) was used to visualize the final tree.

### Comparative Analyses of the RdRp Gene Sequences Between *G. delicatula* Viruses

Degenerated primers, RdRp_F (TCTTCGTATGCCAGCACAACT) and RdRp_R (WAGAGCTCCATGAATCATYCC), were designed based on the RdRp regions of GdelRNAV-01 and of Csp03RNAV that infects *Chaetoceros* sp. strain SS08-C03 (AB639040), using Geneious 9.1.3 (Biomatters Ltd, NZ). These primers were used to amplify about 500 bp of the RdRp domains of GdelRNA-02, GdelRNA-03 and GdelRNA-04. The PCR reaction mixture (25 μL final volume) consisted of 1× Platinum *Taq* buffer (final concentration, Invitrogen), 2 mM MgCl_2_, 0.2 mM dNTP, 1 μM of each primer, 2U of Platinum *Taq* and 1 μL of cDNA. PCR amplifications were performed with the following conditions: an initial incubation step at 94°C for 75 s, followed by 40 cycles of denaturation at 94°C for 45 s, annealing step at 56°C for 45 s and extension at 72°C for 1 min. The 40 cycles were followed by a final extension step at 72°C for 9 min. PCR products were sent to Fasteris (https://www.fasteris.com/dna/, Plan-les-Ouates, Switzerland) for an enzymatic purification and for Sanger Sequencing using the degenerated primers RdRp_F and RdRp_R. The sequence alignment was generated by the MAFFT version 7.222 available on Geneious 9.1.3 (Biomatters Ltd, NZ).

### Biogeography of GdelRNAV-01 in Natural Environments

To determine the distribution of GdelRNAV-01, environmental sequences data were downloaded from public databases and bioinformatics workflows were designed under Galaxy instance of the ABIMS platform: http://galaxy3.sb-roscoff.fr (Giardine et al., 2005) in order to search for homologs of GdelRNAV-01 genome sequences. Briefly, when necessary, data were trimmed and quality filtered and reads were mapped against GdelRNAV-01 genome using the Bowtie2 tool with default parameters (Langmead and Salzberg, 2012). In some cases, the GdelRNAV-01 genome or RdRp domain was directly blasted against environmental sequences using Geneious 9.1.3. This was the case for sequences obtained by Culley et al. (2003, 2006, 2007) and Culley and Steward (2007) with data volume <200 sequences.

### Accession Numbers

The nucleotide sequences of the GdelRNAV-01 genome, GdelRNAV-02, GdelRNAV-03, and GdelRNAV-04 RdRp domains were deposited in the NCBI database under accession number MH706768, MH706769, MH706770 and MH706771, respectively.

Sequences obtained from the eukaryotic nuclear rRNA/ITS for *G. delicatula* strains were also deposited in the NCBI database: RCC3083 (MH712327), RCC4834 (MH712328), RCC5777–RCC5789 (MH712329–MH712341) for the 18S, RCC3083 (MH712342), RCC4834 (MH712343), RCC5777–RCC5787 (MH712344–MH712354) for the partial 28S and RCC3083 (MH714686), RCC4834 (MH714687), RCC5777–RCC5788 (MH714688–MH714699) for the ITS gene marker.

## Results

### *In situ G. delicatula* Dynamics and Cultural Diversity

**Figure 1:**
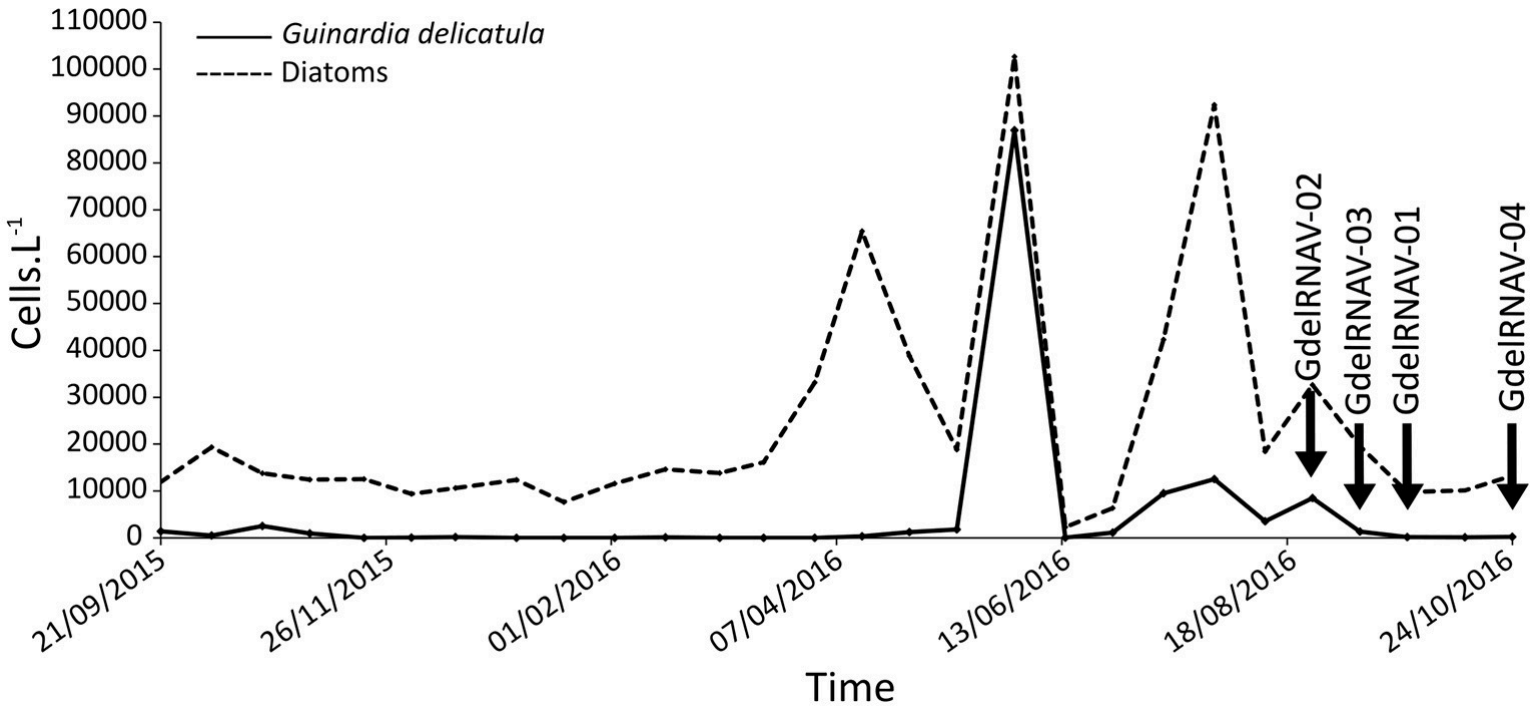
Temporal dynamics of *Guinardia delicatula* (solid line) and all diatoms (dash line) at SOMLIT-Astan station (Western English Channel) during the September 2015–October 2016 period. All along this period a protocol designed for the isolation of viruses lytic to *G. delicatula* was applied to seawater samples collected every fortnight. The arrows point to sampling dates on which the virus strains GdelRNAV-01 to 04 were successfully isolated.

During the sampling course (Sept 2015–Oct 2016), we recorded several blooming episodes of *G. delicatula*. A first small peak (2,560 cells·L^−1^) was detected in October 2015 (Figure 1). In 2016, the diatom bloom that occurred mid-June was dominated by *G*. *delicatula* (86,960 cells·L^−1^, 84.7% of the total diatom counts) while two smaller peaks were observed during the end of summer (12,540 cells·L^−1^ on the 28th of July, 8,500 cells·L^−1^ on the 26th of August).

During the sampling period, a total of 13 new *G*. *delicatula* strains were successfully isolated and maintained in culture (RCC5777-RCC5790, Table 1). Sequencing of the SSU-18S, ITS, and partial LSU-28S gene markers revealed very low variability among strains. For the 18S rDNA gene, the 1,655 bp alignment of the 14 strains indicated 100% of identity between sequences. The ITS gene sequences (646 bp) of 11 of the strains studied were identical while the sequences of RCC4834 (MH714687) and 5783 (MH714694) differed respectively by 2 and 1 position. Concerning the 28S D1-D3 region, 11 strains had identical sequences (833 bp) while a deletion was detected in the RCC5780 sequence (MH712347). The 28S D1-D3 region of strains RCC5788 and RCC5789 were not sequenced (strains lost). Strain RCC5790 was lost before amplification by all three gene markers.

### Viral Isolation

**Figure 2:**
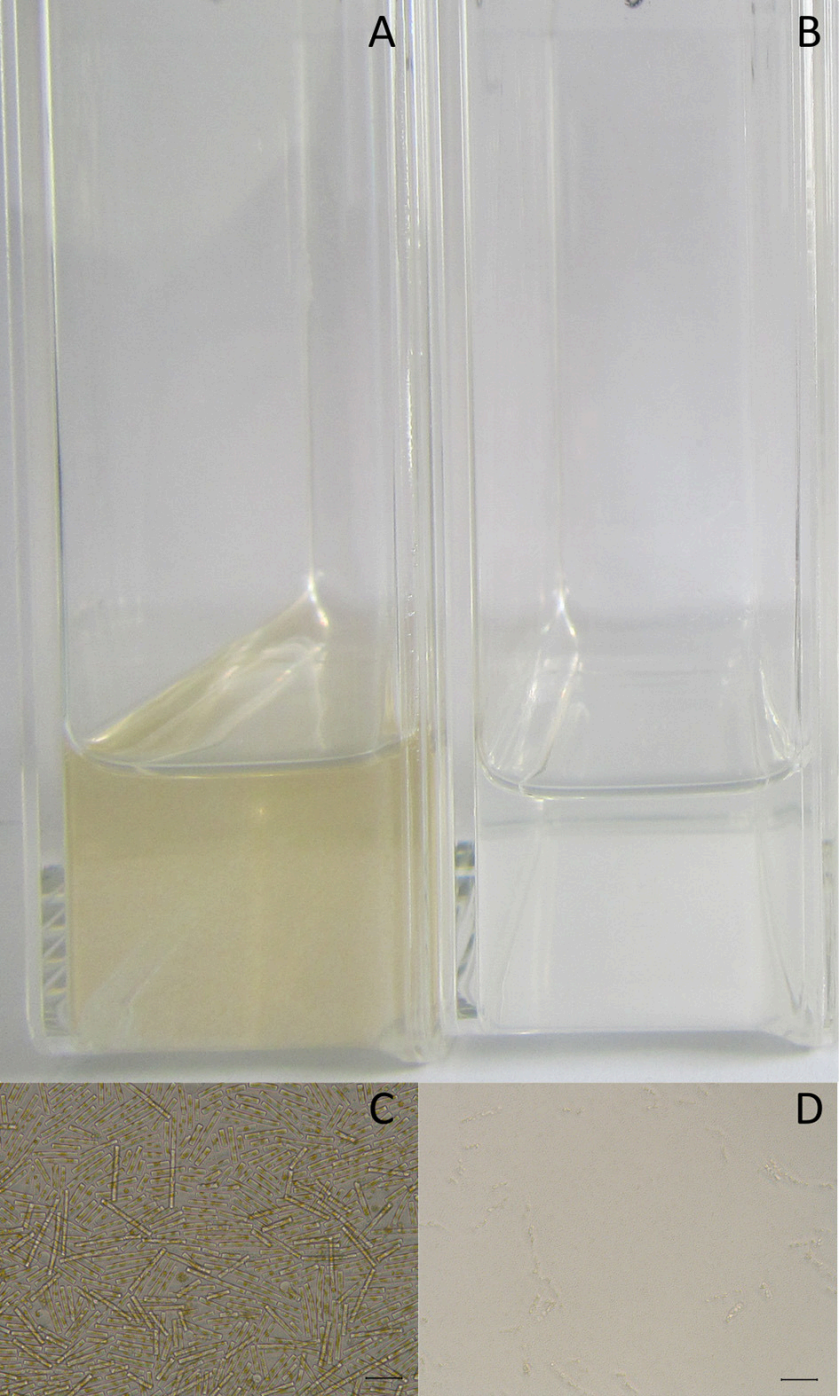
Aspect of healthy cultures of *G. delicatula* RCC3083 **(A,C)** and infected cultures by GdelRNAV-01 **(B,D)** that show disintegration of host cells. **(A,B)** Pictures of the flasks, **(C,D)** Light microscopy micrographs showing heathy cells with golden brown plastids **(C)** or cells totally degraded **(D)**. All pictures were taken 14 days post-infection. Scale bars on pictures **(C,D)** 50 μm.

Viruses lytic to *G. delicatula* RCC3083 were isolated between end August 2016 and end October 2016. During this period, four clonal viral strains were successfully isolated and maintained in culture. The inoculation of these viral isolates into fresh host cultures caused the clearance of infected cultures after 2 weeks and led to complete cell degradations (Figure 2).

### Host Ranges

Cross infection experiments, using 15 phytoplankton species, indicated that *G*. *delicatula* was the only species lysed by the four viral isolates. However, all viruses showed clear strain specificity patterns (Table 1). Besides their isolation host (RCC3083), the 4 viruses infected strains RCC5782, RCC5783, RCC5784, and RCC5787 (isolated between October 2015 and September 2016). GdelRNAV-01 was the only virus able to cause lysis of *G*. *delicatula* RCC5785 (isolated in May 2016). For some host-virus combinations, lysis was not complete (cells with plastids observed in addition to empty frustules 14 dpi). Due to a boarder host range, GdelRNAV-01 that was isolated on the 23rd of September 2016, was chosen for detailed morphological, physiological and genetic characterization.

### Morphological Features of Infected Cells and GdelRNAV Particles

**Figure 3:**
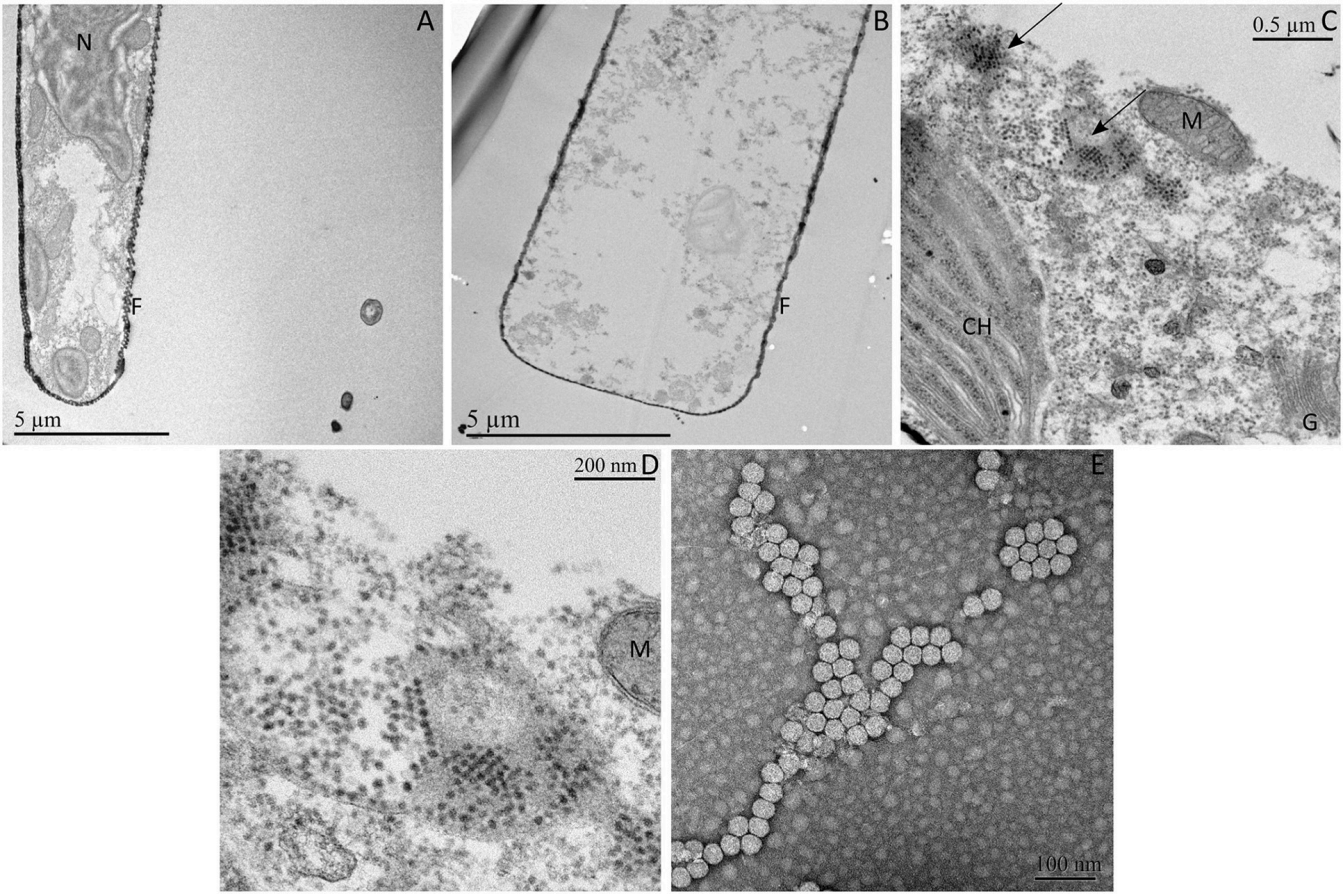
Ultrathin sections of *Guinardia delicatula* RCC3083 and negatively stained GdelRNAV-01 particles obtained by TEM. **(A)** Healthy control. **(B–E)** *G*. *delicatula* infected by GdelRNAV-01 at 72 hpi. **(C)** Crystalline arrays and dispersed viral particles accumulated in the host cytoplasm. **(D)** Higher magnification of panel C of GdelRNAV-01 in the host cytoplasm. **(E)** Negatively stained GdelRNAV-01 particles. Arrows: Crystalline arrays. F, frustule; G, Golgi apparatus; M, mitochondrion; N, nucleus, CH, chloroplast.

Thin sections of *G*. *delicatula* cells showed clear signs of degradation of the cell ultrastructure (few remaining organelles, dispersed traces of cytoplasm) 72 h after the inoculation of GdelRNAV-01 compared to a healthy host (Figures 3A,B). GdelRNAV-01 accumulates in the host cytoplasm where it forms both crystalline arrays and unordered groups of particles (Figures 3C,D). No viral particle was observed in the control (Figure 3A).

The TEM examination of GdelRNAV-01 progenies revealed untailed particles of 35 ± 2 nm in diameter (*n* = 173) with a hexagonal outline suggesting an icosahedral symmetry and the absence of outer membrane (Figure 3E).

Virions of GdelRNAV-02, GdelRNAV-03, and GdelRNAV-04 displayed the same morphological features as GdelRNAV-01 with particles diameter of 38 ± 2 nm (*n* = 98), 36 ± 2 nm (*n* = 105) and 38 ± 1.5 nm (*n* = 97), respectively (data not shown).

### Infection Dynamic of GdelRNAV-01

**Figure 4:**
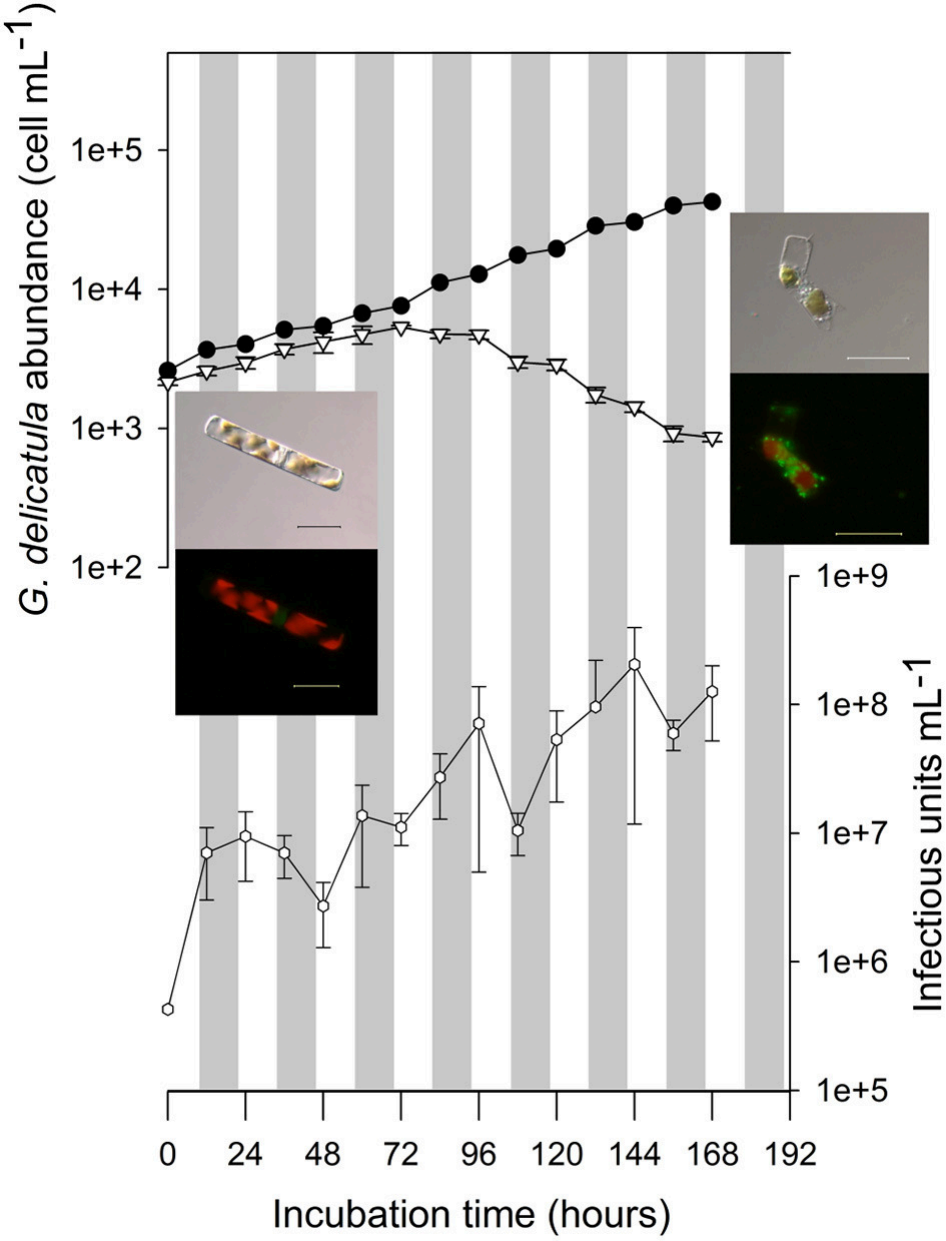
Infection kinetic of *Guinardia delicatula* RCC3083 by GdelRNAV-01. Abundances of diatom hosts in the control culture (black circles) and in infected cultures (open triangles) were obtained using optical microscopy. Viral titers (open hexagons) were estimated using the MPN method. Error bars were estimated based on counts obtained in triplicates of infected cultures. Gray rectangles represent the dark phases. Pictures obtained using transmitted-light and epifluorescence microscopy illustrate the morphology of *G. delicatula* cells in control and infected cultures at T0 and Tfinal. With epifluorescence microscopy the red natural fluorescence of chloroplasts and green fluorescence of PicoGreen stained nucleic acids are observed. At Tfinal, the green fluorescence is due to the presence of bacteria. Scale bars: 20 μm.

After inoculation of GdelRNAV-01 in cultures of *G. delicatula* strain RCC3083, infected cells grew exponentially as in control cultures until 72 h post-inoculation (5,344 cell·mL^−1^ in infected cultured, Figure 4). Cell morphology was similar in infected and control cultures (cells forming colonies with amoeboid-shaped chloroplasts) (Figure 4, optical and epifluorescence micrographs). Then, diatom cell abundance decreased rapidly in infected cultures, with a stabilization step between night and day measurements. At the end of the experiment (168 h), diatom abundance in infected cultures reached 860 cell·mL^−1^ (mean of the three replicates) and was lower than at T0 (2,140 cell·mL^−1^). Nuclei and chloroplasts showed signs of degradation (rounded-shaped chloroplasts), and broken frustules heavily colonized by bacteria were observed (Figure 4, optical and epifluorescence pictures). In comparison, diatom cells in control culture exhibited an exponential growth during all the experiment.

The first increase of viral titer occurred at 12 hpi, suggesting that the latent period is shorter than 12 h. Periods of increase in virus titer alternated with periods of stagnation suggesting multiple cycles in spite of the high MOI (multiplicity of infection).

The burst size, calculated as the number of viral particles produced per host cell, for a given period, was estimated to be 9.34 × 10^4^ infectious units·cell^−1^.

### Stability of the Viral Particles

The viral suspension of GdelRNAV-01 has been exposed during 24 h to a broad range of temperatures (Table 2). The virus remained infectious from −196 to 45°C showing a high thermal stability. No viral lysis was recorded above 50°C.

**Table 2:** GdelRNAV-01 sensitivity to thermal and solvent treatments.

**Treatments**	**Sensitivity**
**TEMPERATURE (°C)**	
−196	–
−80	–
−20	–
4	–
10	–
18	–
25	–
30	–
40	–
45	–
50	+/–
55	+
60	+
**CHLOROFORM (%)**	
10	–
50	–

–*, no loss of viral infectivity; +, loss of infectivity; +/–, partial loss of infectivity (healthy host cells still present in the wells 14 days post-inoculation)*.

GdelRNAV-01 was not susceptible to chloroform since no loss of viral infectivity was reported regardless of the chloroform concentration (Table 2).

### GdelRNAV-01 Genome

**Figure 5:**
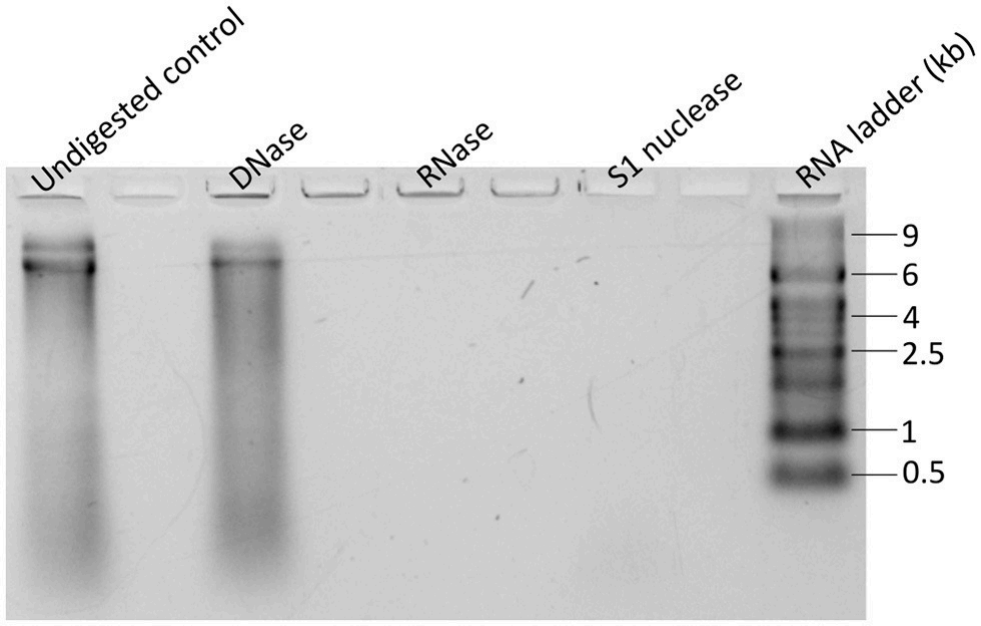
Nucleic acids type of GdelRNAV-01 after extraction. Extracts were treated with DNase treatment, with RNase treatment, or with S1 nuclease treatment.

Gel electrophoresis of purified GdelRNAV-01 nucleic acids and enzymatic digestions tests with DNase, RNase, and S1 Nuclease, indicated that GdelRNAV-01 possesses a single-stranded RNA genome of about 9 kb (Figure 5). Comparable results were obtained for GdelRNAV-02, GdelRNAV-03 and GdelRNAV-04 (data not shown), implying that these viruses possess a single-stranded RNA genomes of 9 kb.

The assembled genome of GdelRNAV-01 was 9,233 nt in length, excluding a poly(A) tail. The adenine, cytosine, guanine and uracil richness were estimated to be 30.1, 17.6, 19.3, and 33%, respectively.

**Figure 6:**
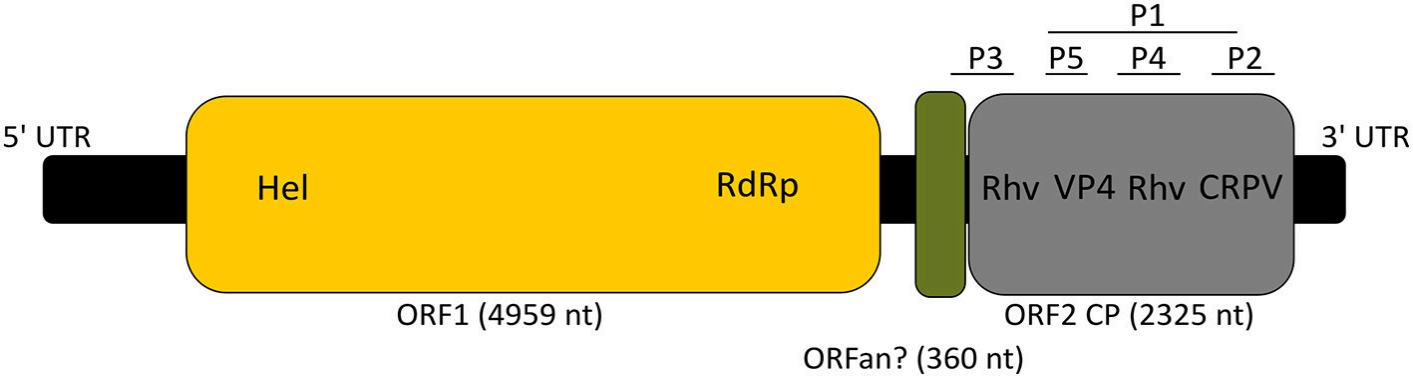
Schematic genome organization of GdRNAV-01 (9,233 nt). 5′UTR: 5′ untranslated region (1,008 nt), 3′ UTR: 3′ untranslated region (367 nt). The yellow box indicates the replication polyprotein with Hel: Helicase and RdRp: RNA-dependent RNA polymerase. The gray box represents the capsid proteins (CP) with domains corresponding to the Rhv_like superfamily interspaced by the Dicistro_VP4, and the CRPV_capsid superfamily. The green box indicates the possible ORFan. P1 to P5: structural proteins. Segments corresponding to P1 to P5 are not scaled on the genome sequence.

The GdelRNAV-01 genome consisted of a 5′ untranslated region (UTR, 1,008 nt), two ORFS separated by an intergenic region (IGR, 574 nt) and a 3′ UTR of 367 nt (Figure 6). The size 5′ and 3′ UTR may not be completed as we did not do RACE. The first ORF was 4,959 nt long, representing 53.7% of the whole genome. It clustered two replication-related proteins: a helicase domain (110 amino acids) and a RNA-dependent RNA polymerase (RdRp) domain (291 amino acids) (Figure 6). The BLAST searches (Table 3) revealed that both proteins were closely related to *Chaetoceros* sp. RNA virus 03, *Chaetoceros tenuissimus* RNA virus type-II, to Marine RNA virus JP-A (Culley et al., 2007), and to Beihai picorna-like viruses and Wenzhou picorna-like virus 50, that infect invertebrates (Shi et al., 2016).

**Table 3:** Best hits from BLASTP results showing significant alignments with the helicase and RdRp domains and the ORF-2 of GdelRNAV-01.

	**Score**	**Query cover**	***E*-value**	**Identity**	**Accession number**
**HELICASE BLASTP BEST HITS**					
Beihai picorna-like virus 4	225	100%	4E-66	98%	YP_009333566.1
*Chaetoceros* sp. RNA virus 3	225	100%	4E-66	98%	BAK40203.1
Wenzhou picorna-like virus 50	190	100%	8E-54	76%	APG78567.1
Marine RNA virus JP-A	182	100%	4E-51	71%	YP_001429581.1
Beihai picorna-like virus 1	176	100%	7E-49	72%	YP_009333509.1
**RdRp BLASTP BEST HITS**					
*Chaetoceros* sp. RNA virus 03	585	100%	0	93%	BAK40203.1
Beihai picorna-like virus 4	582	100%	0	92%	YP_009333566.1
Wenzhou picorna-like virus 50	490	100%	2E-158	76%	APG78567.1
Beihai picorna-like virus 1	491	100%	5E-158	76%	YP_009333509.1
Marine RNA virus JP-A	481	100%	1E-154	74%	YP_001429581.1
*Chaetoceros tenuissimus* RNA virus type-II, isolate SS10-45V	411	95%	8E-129	68%	BAP99822.1
*Chaetoceros tenuissimus* RNA virus type-II, isolate SS10-39V	411	95%	9E-129	68%	BAP99820.1
*Chaetoceros tenuissimus* RNA virus type-II, isolate SS10-16V	411	95%	1E-128	68%	YP_009111336.1
**ORF2 BLASTP BEST HITS**					
Beihai picorna-like virus 4	1,088	97%	0	70%	YP_009333567.1
*Chaetoceros* sp. RNA virus 03	1,034	89%	0	73%	BAK40204.1
Marine RNA virus JP-A	904	99%	0	55%	YP_001429582.1
CtenRNAV type-II	894	100%	0	58%	YP_009111337.1
CtenRNAV SS10-39V	893	100%	0	58%	BAP99821.1
CtenRNAV SS10-45V	892	100%	0	58%	BAP99823.1
Beihai picorna-like virus 1	820	90%	0	57%	YP_009333510.1

The second ORF (2325 nt, 25.2% of the viral genome) encoded for putative structural proteins of the capsid based on the detection of 4 conserved capsid domains. This ORF contained two domains that belong to the Picornavirus capsid protein domain_like (Rhv1 and 2), one to the Cricket paralysis virus VP4 domain from the *Dicistroviridae* family (Dicistro_VP4) and the last domain shared significant homology with the cricket paralysis virus (CRPV) capsid protein like (Figure 6). As with the first ORF, best hits of ORF2 using BLASTP (774 amino acids) corresponded to sequences of ssRNA viruses (Table 3).

In the IGR, a small conserved region was detected (360 nt, 4% of the whole genome). BLASTn searches revealed two hits belonging to *Chaetoceros* sp. RNA virus 03 (*E*-value: 2e-88) and Beihai picorna-like virus 4 (*E*-value: 5e-54). However, no statistically significant matching protein sequence was found using BLASTP searches (119 amino acids).

### Structural Proteins

**Figure 7:**
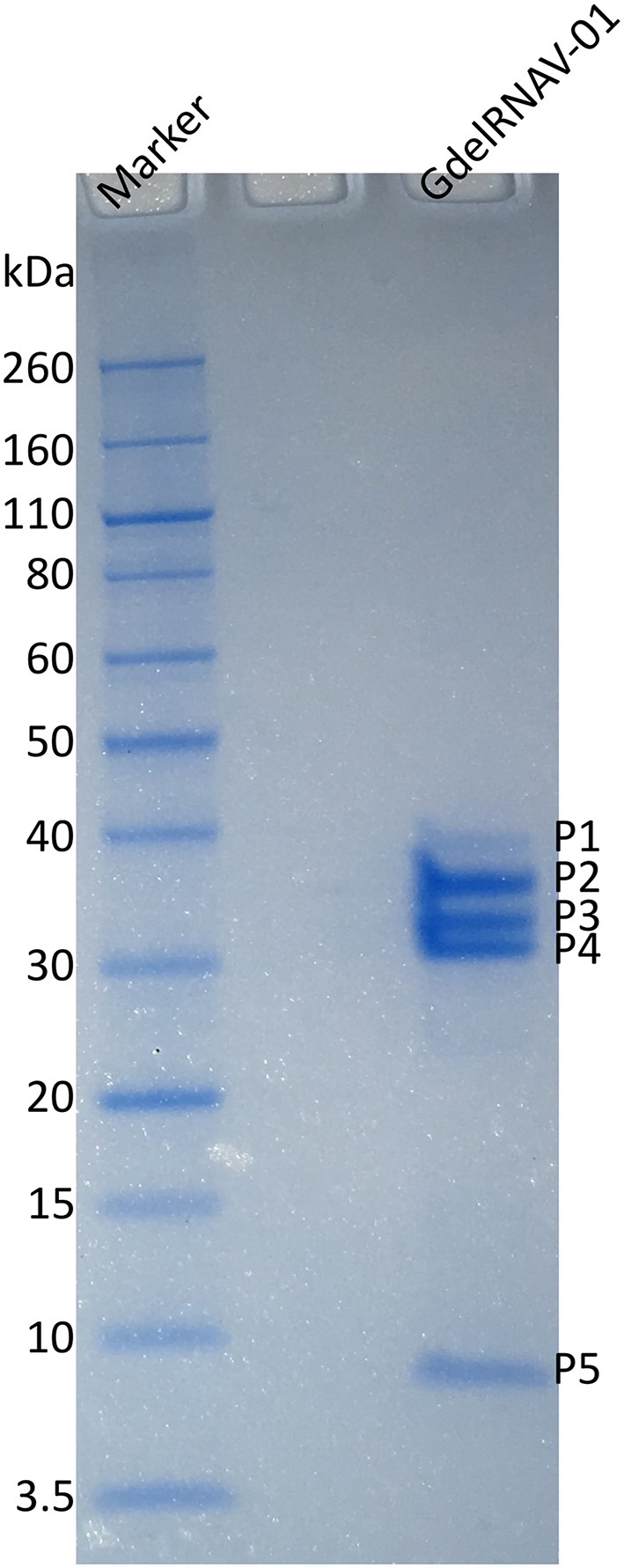
Analysis of the structural proteins of GdelRNAV-01 using SDS-PAGE. Lane Marker: Novex sharp unstained protein standard marker (kDa). Lane GdelRNAV-01: Denatured proteins of purified GdelRNAV-01. P1 to P5 represent the proteins 1 to 5.

The SDS PAGE showed five proteins of variable staining intensity (Figure 7). Four proteins of respectively 33.9, 29.8, 27, and 6.8 kDa (P2, P3, P4, and P5, respectively) were intensively stained while the largest protein of 38.6 kDa (P1) had a weaker intensity (Figure 7). The amino acid sequences of each protein analyzed by mass spectrometry (MS) were found in the predicted sequence of the ORF2 (Figure 6 and Tables S1, S2). The smallest protein (P5) (6.8 kDa predicted from the gel and 4.7 kDa from the amino acid sequence) corresponded to the Dicistro_VP4 domain. The predicted peptides of P2 (33.9 kDa on the SDS-PAGE gel, 16.8 kDa based on the amino acid sequence) matched the C-ter region of the CRPV domain. The protein P4 was more central and peptides analyzed by MS corresponded to the N-terminal region of Rhv2 domain. MS analysis of P3 peptides revealed that they matched the upstream region of ORF2 up to Rhv1 domain. The largest protein P1 (38.6, 51.7 kDa predicted) encompassed VP4, Rhv2, and CRPV domains.

### Phylogenetic Analysis of the *Picornavirales*

**Figure 8:**
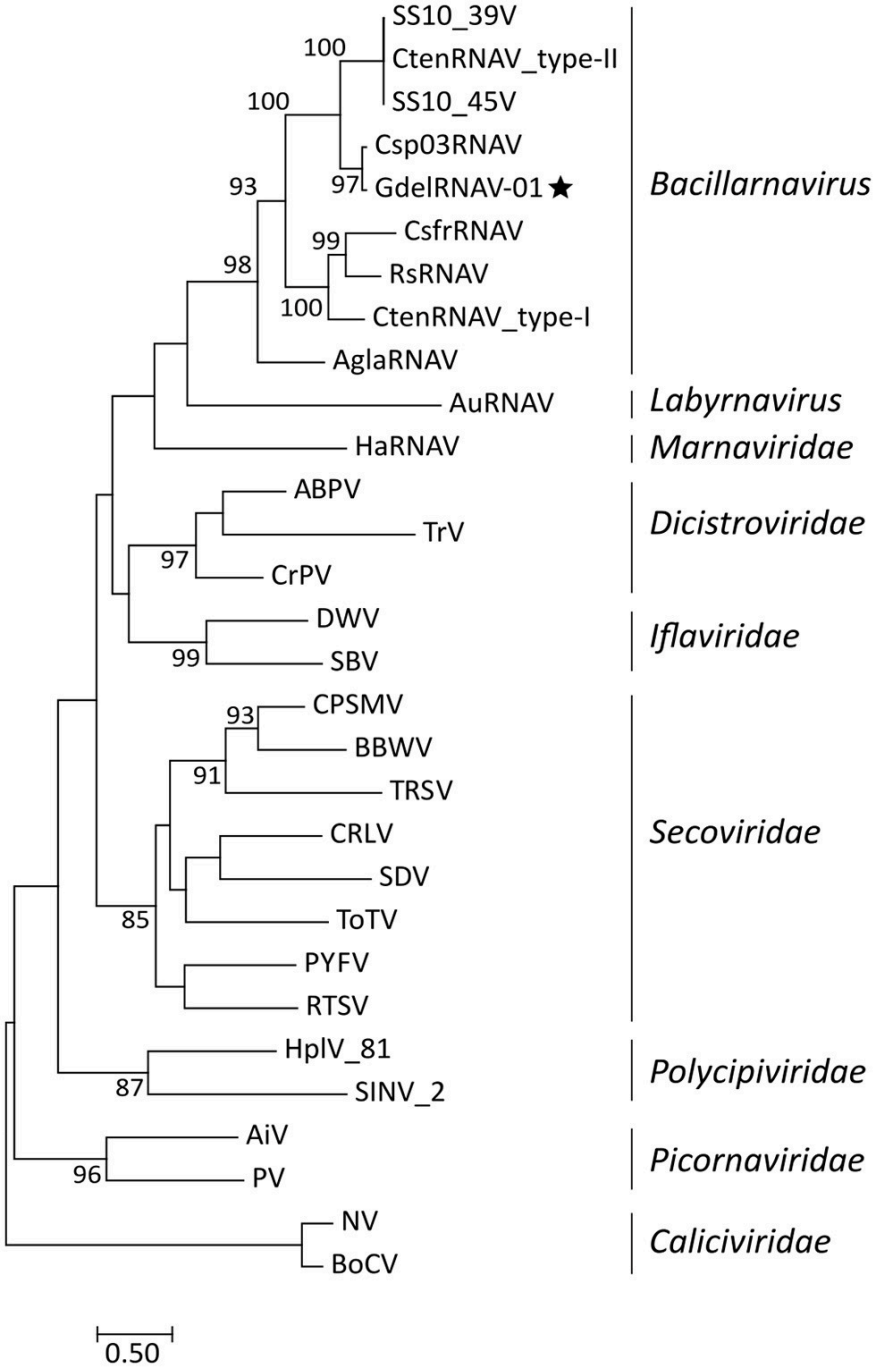
Phylogenetic rooted tree based on RdRp sequences of representative viruses from the *Picornavirales* order. *Caliciviridae* viruses were taken as outgroup. The star indicates the position of GdelRNAV-01 in the genus *Bacillarnavirus*. The Maximum Likelihood tree was generated using PhyML 3.0 with 1,000 replications and a LG + G + I + F substitution model according to the SMS analyses. Bootstraps values (%) >80 are shown. Scale bar indicates the number of substitutions per site. Virus abbreviations: ABPV, acute bee paralysis virus NC_002548.1; AiV, Aichi virus, AB010145; AglaRNAV, *Asterionellopsis glacialis* RNA virus NC_024489; AuRNAV, *Aurantiochytrium* single-stranded RNA virus), BAE47143; BoCV, Bovine enteric calicivirus, AJ011099; BBW, broad bean wilt virus 1 NC_005289.1; CsfrRNAV, *Chaetoceros socialis* f. radians RNA virus, AB469874; Csp03RNAV, *Chaetoceros* sp. number03 RNA virus, AB639040; CtenRNAV type-I, *Chaetoceros tenuissimus* RNA virus, AB375474; CtenRNAV type-II, AB971661; CtenRNAV_SS10V-39V, AB971662; CtenRNAV_SS10V-45V, AB971663; CRLV, cherry rasp leaf virus, NC_006271.1; CPSMV, cowpea severe mosaic virus, NC_003545; CrPV, cricket paralysis virus, NC_003924; DWV, deformed wing virus, NC_004830; HaRNAV, *Heterosigma akashiwo* RNA virus, NC_005281; HplV-81, Hubei picorna-like virus 81 strain CJLX25805, KX884540.1; HplV-82, Hubei picorna-like virus 82 KX883688.1; PV, human poliovirus 1 Mahoney, V01149; IFV, infectious flacherie virus NC_003781.1; NV, Norwalk virus, M87661; PYFV, Parsnip yellow fleck virus, D14066; RsRNAV, *Rhizosolenia setigera* RNA virus, AB243297; RTSV, rice tungro spherical virus, AAA66056; SBV, sacbrood virus, NC_002066; SDV, Satsuma dwarf virus RNA 1 NC_003785.2; SINV-2, *Solenopsis invicta* virus 2 EF428566.1; TRSV, tobacco ringspot virus RNA 1 NC_005097.1; ToTV, tomato torrado virus RNA 1 NC_009013.1; TrV, triatoma virus, NC_003783.

Phylogenetic reconstructions based on the analysis of the RdRp amino acid sequences of a selection of *Picornavirales* revealed that GdelRNAV-01 clusters among the monophyletic genus *Bacillarnavirus* (Figure 8). Sequences of these viruses, that infect diatom species, gathered in a clade supported by a high bootstrap value (98%). GdelRNAV-01 was most closely related to *Chaetoceros* sp. number03 RNA virus (Csp03RNAV), a virus infecting the marine diatom *Chaetoceros* sp. (Tomaru et al., 2013a).

### Intraspecific Comparison of GdelRNAV Viruses

Nucleotide partial sequences of the RNA-dependent RNA polymerase (RdRp) of GdelRNAV-01, GdelRNAV-02, GdelRNAV-03 and GdelRNAV-04 were highly similar, with 474 identical sites on 478 bp, representing 99.2% of identity (Figure S1). The slight differences between these four RdRp sequences are shown Figure S1 in red frames.

### Distribution of GdelRNAV-01 in Natural Environments

Environmental surveys allowed us to study the natural distribution of GdelRNAV-01 across marine and fresh water environments (Table 4). In total, 18,858 homologous reads (488.2 bp on average) mapped against the GdelRNAV-01 RdRp gene marker. They were exclusively found in temperate coastal water stations off British Columbia. At these stations, deep-sequencing of the RdRp has been carried out to assess the diversity and composition of the ssRNA viral community (Gustavsen et al., 2014).

**Table 4:** Results of the mapping of the GdelRNAV-01 genome or gene sequences onto environmental data.

**References**	**Database**	**Accession number**	**RdRp amplicons/** **Metagenomes**	**Sampling site**	**Region**	**Number of positive reads mapping**	**Length (bp) min–mean–max**
Culley et al., 2003	GenBank	AY285747–AY285767	RdRp domain	British Columbia, Canada	Pacific temperate coastal waters	0	0
Culley et al., 2006	NCBI BioProject	PRJNA17367	Genome	British Columbia, Canada	Pacific temperate coastal waters	0	0
Culley et al., 2007	GenBank	NC_009756–NC_009758	Genome	British Columbia, Canada	Pacific temperate coastal waters	0	0
Culley and Steward, 2007	GenBank	EF591792–EF591815	RdRp domain	Hawaii and California, USA	Pacific subtropical waters	0	0
Rosario et al., 2009	NCBI BioProject	PRJNA36649	Genome	Manatee County, Florida, USA	Reclaimed waters	0	0
Djikeng et al., 2009	MetaVir	1,153 and 1,154	Genome	Maryland, USA	Lake, freshwaters	0	0
Culley et al., 2014	GenBank	KC620972–KC621051	RdRp domain	Hawaii, USA	Pacific tropical waters	0	0
Culley et al., 2014	iMicrobes	CAM_SMPL_000815 and CAM_SMPL_000824	Genome	Hawaii, USA	Pacific tropical waters	0	0
Gustavsen et al., 2014	NCBI BioProject	PRJNA267690	RdRp domain	British Columbia, Canada	Pacific temperate coastal waters	18,858	241–488, 2–508
Lõpez-Bueno et al., 2015	Metavir	4,488–4,490	Genome	Livingston Island, Antarctic	Lake, freshwaters	0	0
Miranda et al., 2016	NCBI BioProject	PRJNA266680	Genome	Western Antarctic Peninsula	Antarctic polar waters	0	0

## Discussion

The marine diatom *G. delicatula* is a cosmopolitan species that dominates seasonal blooms in the English Channel and the North Sea. In the environment, this species is known to be infected by several eukaryotic parasites. In this study, we described for the first time viruses that infect *G*. *delicatula*, and probably contribute to the control of its bloom dynamics.

Morphological and genomic analyses indicated that the new *G*. *delicatula* viruses isolated during this study belong to the unassigned genus *Bacillarnavirus* within the order *Picornavirales* (Tomaru et al., 2015). This genus includes ssRNA viruses that infect diatoms and includes three species to date (ICTV). Like other members of the unassigned genus *Bacillarnavirus*, virions are small naked particles (35 nm in diameter) with a hexagonal outline, suggesting an icosahedral symmetry, and they contain a positive-sense ssRNA genome. During the infection, viral progenies accumulate in the host cytoplasm, where they form both crystalline arrays and unordered particles, before their release by cell lysis. The genome architecture of GdelRNAV-01 is similar to that of other *Picornavirales* (Koonin et al., 2008). The 9 kb genome of GdelRNAV-01 comprises 2 ORFs, coding respectively for replication and structural polyproteins with best hits to sequences of ssRNA viruses of the diatom *Chaetoceros* spp., as well as marine environmental virus genomes (Culley et al., 2007) and viral sequences assembled from transcriptomics (Shi et al., 2016). More precisely, the first ORF includes domains coding for the RNA-dependent RNA-polymerase (RdRp) as well as a helicase. The RdRp is traditionally used as a diversity marker for RNA viruses (Koonin et al., 1993; Culley et al., 2003). The sequencing of this gene showed low genetic variability between the four GdelRNAV isolates, suggesting that the four strains belong to the same virus. The second ORF encodes for the structural polyprotein showing the same conserved protein domains (Rhv_like, Dicistro_VP4, CRPV_capsid) and architecture as the other diatom viruses. According to our proteomic analyses, a large protein (P1), whose amino acid sequence appeared to overlap that of the three protein domains, was detected. This protein may correspond to an immature form associated to provirions. It is likely that this precursor capsid protein cleaves into smaller proteins after a maturation process as described or speculated for other members of the order *Picornavirales* (Lang et al., 2004; Mullapudi et al., 2016). Apart from this putative immature protein, our MS results suggest that GdelRNAV virions are constituted of four structural proteins that matched with each of the four conserved domains predicted in the genome sequence. Interestingly, other known members of *Bacillarnavirus* display only three structural proteins (Tomaru et al., 2015).

The functional characterization showed that GdelRNAV is strain specific, virions are produced rapidly (in our case, latent period <12 h) and infection ultimately induced host mortality through cell lysis, as reported for other algal viruses including both RNA and dsDNA viruses (for review see Brussaard and Martínez, 2008 and reference therein). For GdelRNAV, the estimated burst size reached 9.34 × 10^4^ virions per host cell, which is higher than reported values for other ssRNA viruses [<100–10^4^ virions per cell (Tomaru et al., 2015)]. One divergent feature of ssRNA diatom viruses compared to known algal viruses is the simultaneous increase in host and viral concentrations during the first days of incubation (72 h in our case) and the occurrence of multiple viral cycles (Shirai et al., 2008; Tomaru et al., 2009, 2011b, 2014; Kimura and Tomaru, 2013). A theoretical calculation suggests that only 3.3% of *G*. *delicatula* cells produced viral progenies at the initial time of the kinetics and that the percentage of permissive cells increases along the growth curve (see Table S3). It is thus likely that the host cell culture, although clonal, exhibited different degrees of viral susceptibility.

Diverse mechanisms of host resistance to viral infection have been described in marine microalgae. For example, in the picoeukaryote *Ostreococcus tauri*, some proteins encoded in chromosome 19 were shown to be involved in the host defense against viral attack (Yau et al., 2016). Unfortunately, *G*. *delicatula*'s genome sequence is not available to speculate on similar mechanisms operating in our virus-host model system. However, previous studies demonstrated that the physiological status of diatom host cells can also determine the outcome of viral infection. *Chaetoceros* host population generally became more permissive to viral infection with the progression of the stationary growth (Tomaru et al., 2014). This led to the hypothesis that diatoms with a high growth rate may tolerate viral infection while cells with less vigorous growth rate undergo rapid lysis and do not participate to the bloom formation (Tomaru et al., 2015). Interestingly, we attempted to isolate *G*. *delicatula* viruses throughout the year but isolations were successful only with samples collected in late summer. We cannot rule out that the host strain used for viral isolation was not permissive to the spring and summer viral populations. Yet, the amplitude of *G*. *delicatula* late summer bloom is consistently lower compared to spring and early summer blooms at SOMLIT-Astan (our study and RESOMAR Pelagos database). It is tempting to speculate that the late summer environmental conditions were less favorable for the growth of *G*. *delicatula*, which, in turn, may have been more vulnerable to viral attack. *G*. *delicatula* is also known to be infected by diverse parasites, such as *Pirsonia* (Kühn et al., 1996) and *Cryothecomonas* (Drebes et al., 1996). *G*. *delicatula* blooms may thus be controlled by a complex network of pathogens, among which viruses may not be the primary cause of bloom disintegration, as already reported for *Chaetoceros* spp. (Tomaru et al., 2011a, 2018). In any case, variability in viral susceptibility of the host is probably contributing to the sustainability of these diatom bloom events in natural habitats.

Isolating and characterizing new viruses infecting ecologically relevant hosts is a prerequisite to advance our understanding of the large amount of environmental sequences collected worldwide. Data-mining of RNA viromes that are publicly available showed that the genome of GdelRNAV-01 recruited homologs in environments where *G*. *delicatula* is known to develop [based on Ocean Biogeographic Information System (OBIS) database and Hobson and McQuoid (1997)]. Although very few RNA viromes are available, these preliminary results suggest that GdelRNAV occur in different temperate coastal waters. Seasonal metagenomic monitoring in the Western English Channel should be considered to investigate the composition, the prevalence and the temporal dynamics of this relevant virus–host model system. It will contribute to have a closer look at the relative contribution of the different pathogens to the control of diatom blooms, necessary to understand the fate of these prominent organisms in marine systems.

## Author Contributions

LA designed and conducted the experiments and analyses and wrote the manuscript. NS designed the study, contributed to the experiments, wrote the manuscript. FR-J helped for sampling and for kinetic experiments, and carried out taxonomic counts in the frame of the Roscoff SOMLIT-Astan time series. FL isolated *Guinardia* hosts, performed the PCR and participated to the analyses of the eukaryotic gene marker. SC designed the genome recruitment analysis. ErC assembled the viral genome. EmC performed and analyzed the proteomics data. EB provided technical support. A-CB designed the study, contributed to the experiments, wrote the manuscript.

### Conflict of Interest Statement

The authors declare that the research was conducted in the absence of any commercial or financial relationships that could be construed as a potential conflict of interest.
