# Supplementary material for: First Viruses Infecting the Marine Diatom Guinardia delicatula
Source: Front Microbiol. 2019 Jan 9;9:3235. doi: 10.3389/fmicb.2018.03235 (PMC6334475; doi:10.3389/fmicb.2018.03235)
Supplement: Supplementary file 3 [file Table_3.docx]

Table S3. Estimated percentages of permissive host cells during the infection kinetics. For each putative lytic cycle the number of permissive cells was calculated by dividing the number of viruses produced (maximum number of viruses minus number of viruses at the beginning of the lytic cycle) by the burst size (see material and methods for calculation of burst size). The % of permissive cells was calculated in relation to the abundance of diatom cells at the beginning of each lytic cycle.

| **Incubation time (hours) corresponding to the beginning of each putative lytic cycle** | **Permissive host cells (%)** |
| --- | --- |
| 0 | 3.3 |
| 48 | 2.8 |
| 72 | 12 |
| 108 | 69.4 |
