# Supplementary material for: First Viruses Infecting the Marine Diatom Guinardia delicatula
Source: Front Microbiol. 2019 Jan 9;9:3235. doi: 10.3389/fmicb.2018.03235 (PMC6334475; doi:10.3389/fmicb.2018.03235)
Supplement: Supplementary file 4 [file Data_Sheet_1.docx]

**Supplemental material and methods**

**Liquid Chromatography−Tandem Mass Spectrometry (LC−MS/MS) Analyses**

Mass spectrometry analysis of the GdelRNAV-01 protein digests were conducted on a LTQ-Orbitrap XL (ThermoFisher Scientific) mass spectrometer. The MS measurements were done with a nanoflow high-performance liquid chromatography (HPLC) system (Dionex,LC Packings Ultimate 3000) connected to a hybrid LTQ-Orbitrap XL (Thermo Fisher Scientific) equipped with a nanoelectrospray ion source (New Objective). The HPLC system consisted of a solvent degasser nanoflow pump, a thermostated column oven kept at 30 °C, and a thermostated autosampler kept at 8 °C to reduce sample evaporation. Mobile A (99.9% Milli-Q water and 0.1% formic acid (v:v)) and B (99.9% acetonitrile and 0.1% formic acid (v:v)) phases for HPLC were delivered by the Ultimate 3000 nanoflow LC system (Dionex, LC Packings). An aliquot of 10 μL of prepared peptide mixtures were loaded onto a trapping precolumn (5 mm × 300 μm i.d., 300 Å pore size, Pepmap C18, 5 μm) over 3 min in 2% buffer B at a flow rate of 25 μL/min. This step was followed by reverse-phase separations at a flow rate of 0.250 μL/min using an analytical column (15 cm × 300 μm i.d., 300 Å pore size, Pepmap C18, 5 μm, Dionex, LC Packings). We ran a gradient from 2−35% buffer B for the first 60 min, 35−60% buffer B from minutes 60−85, and 60−90% buffer B from minutes 85−105. Finally, the column was washed with 90% buffer B for 16 min and with 2% buffer B for 19 min before the next sample was loaded. The peptides were detected by directly eluting them from the HPLC column into the electrospray ion source of the mass spectrometer. An electrospray ionization (ESI) voltage of 1.6 kV was applied to the HPLC buffer using the liquid junction provided by the nanoelectrospray ion source, and the ion transfer tube temperature was set to 200 °C. The LTQ-Orbitrap XL instrument was operated in its data-dependent mode by automatically switching between full survey scan MS and consecutive MS/MS acquisitions. Survey full scan MS spectra (mass range 400−2000) were acquired in the Orbitrap section of the instrument with a resolution of r = 60 000 at m/z 400; ion injection times were calculated for each spectrum to allow for accumulation of 10^6^ ions in the Orbitrap. The ten most intense peptide ions in each survey scan with an intensity above 2000 counts (to avoid triggering fragmentation too early during the peptide elution profile) and a charge state ≥ 2 were sequentially isolated at a target value of 10 000 and fragmented in the linear ion trap by collision-induced dissociation. Normalized collision energy was set to 35% with an activation time of 30 ms. Peaks selected for fragmentation were automatically put on a dynamic exclusion list for 60 s with a mass tolerance of ±10 ppm to avoid selecting the same ion for fragmentation more than once. The following parameters were used: the repeat count was set to 1, the exclusion list size limit was 500, singly charged precursors were rejected, and the maximum injection time was set at 500 and 300 ms for full MS and MS/MS scan events, respectively. For an optimal duty cycle, the fragment ion spectra were recorded in the LTQ mass spectrometer in parallel with the Orbitrap full scan detection. For Orbitrap measurements, an external calibration was used before each injection series ensuring an overall error mass accuracy below 5 ppm for the detected peptides. MS data were saved in RAW file format (Thermo Fisher Scientific) using XCalibur 2.0.7 with tune 2.4. The data analysis was performed with the Mascot Distiller v2.6.1.0 software connected to the Mascot (Mascot server v2.5.01; http://www.matrixscience.com) database search engine for peptide and protein identification using its automatic decoy database search to calculate a false discovery rate (FDR). MS/MS spectra were compared to the GdelRNAV-01 genome sequence. Mass tolerance for MS and MS/MS was set at 10 ppm and 0.5 Da, respectively. The enzyme selectivity was set to full trypsin with one miscleavage allowed. Protein modifications were fixed carbamidomethylation of cysteines, variable oxidation of methionine. Identification results from Mascot (.dat files) were imported into the ProLine Studio software version 1.5 (<http://proline>.profiproteomics.fr). This software was then used to validate protein identification with a peptide rank = 1 and a FDR of 1% on the score at the peptide spectrum matches level. In order to determine the domain of the ORF the most likely to correspond to each detected proteins, quantification of each identified peptide was estimated using spectral count and liquid chromatography peak intensity.
