# Supplementary material for: First Viruses Infecting the Marine Diatom Guinardia delicatula
Source: Front Microbiol. 2019 Jan 9;9:3235. doi: 10.3389/fmicb.2018.03235 (PMC6334475; doi:10.3389/fmicb.2018.03235)
Supplement: Supplementary file 5 [file Image_1.pdf]

```
               1        10        20        30        40        50        60
               |        |         |         |         |         |         |
GdelRNAV-01    CATCAATGCTGCTTTTGCCGCTTTAATAGAAATTGCGGAAAAATGTGGCAGATATACCAA
GdelRNAV-02    CATCAATGCTGCTTTTGCCGCTTTAATAGAAATTGCGGAAAAATGTGGCAGATATACCAA
GdelRNAV-03    CATCAATGCTGCTTTTGCCGCTTTAATAGAAATTGCGGAAAAATGTGGCAGATATACCAA
GdelRNAV-04    CATCAATGCCGCTTTTGCCGCTTTAATAGAAATTGCGGAAAAATGTGGCAGATATACCAA

GdelRNAV-01    TGATGATCTTACGATTATGAGAGGTATTGCAACAGAAATTGCTTATTCATGCGTAGCTTA
GdelRNAV-02    TGATGATCTTACGATTATGAGAGGTATTGCAACAGAAATTGCTTATTCATGCGTAGCTTA
GdelRNAV-03    TGATGATCTTACGATTATGAGAGGTATTGCAACAGAAATTGCTTATTCATGCGTAGCTTA
GdelRNAV-04    TGATGATCTTACGATTATGAGAGGTATTGCAACAGAAATTGCTTATTCATGCGTAGCTTA

GdelRNAV-01    TAATGGAGATATTATTATCCATAAAGGATCAAATCCATCAGGACAAAATTTAACGGTATA
GdelRNAV-02    TAATGGAGATATTATTATCCATAAAGGATCAAATCCATCAGGACAAAATTTAACGGTATA
GdelRNAV-03    TAATGGAGATATTATTATCCATAAAGGATCAAATCCATCAGGACAAAATTTAACGGTATA
GdelRNAV-04    TAATGGAGATATTATTATCCATAAAGGATCAAATCCATCAGGACAAAATTTAACGGTATA

GdelRNAV-01    TATTAACTGTCTCGTTAATTCTTTGCTATTAAGATGTGCATATTTTCACCTTTGGCCTAA
GdelRNAV-02    TATTAACTGTATCGTTAATTCTTTGCTATTAAGATGTGCATATTTTCACCTTTGGCCTAA
GdelRNAV-03    TATTAACTGTATCGTTAATTCTTTGCTATTAAGATGTGCATATTTTCACCTTTGGCCTAA
GdelRNAV-04    TATTAACTGTATCGTTAATTCTTTGCTATTAAGATGTGCATATTTTCACCTTTGGCCTAA

GdelRNAV-01    ACACCTAGGTCAGCCAAAACCTTTTCGTGAGGTTTGTGCTATTATGACTTATGGTGATGA
GdelRNAV-02    ACACCTAGGTCAGCCAAAACCTTTTCGTGAGGTTTGTGCTATTATGACTTATGGTGATGA
GdelRNAV-03    ACACCTAGGTCAGCCAAAACCTTTTCGTGAGGTTTGTGCTATTATGACTTATGGTGATGA
GdelRNAV-04    ACACCTAGGTCAGCCAAAACCTTTTCGTGAGGTTTGTGCTATTATGACTTATGGTGATGA

GdelRNAV-01    TGTTAAAGGTTCTGTAAAGGAAGGCTATGATTGGTTTAATCATATTTCATATGCTGATTT
GdelRNAV-02    TGTTAAAGGTTCTGTAAAGAAAGGCTATGATTGGTTTAATCATATTTCATATGCTGATTT
GdelRNAV-03    TGTTAAAGGTTCTGTAAAGGAAGGCTATGATTGGTTTAATCATATTTCATATGCTGATTT
GdelRNAV-04    TGTTAAAGGTTCTGTAAAGGAAGGCTATGATTGGTTTAATCATATTTCATATGCTGATTT

GdelRNAV-01    CTTAAGAGAACGTGATATGGTTTTTACTATGCCAGATAAAGAATCTGAACCTACCCCATA
GdelRNAV-02    CTTAAGAGAACGTGATATGGTTTTTACTATGCCAGATAAAGAATCTGAACCTACCCCATA
GdelRNAV-03    CTTAAGAGAACGTGATATGGTCTTTACTATGCCAGATAAAGAATCTGAACCTACCCCATA
GdelRNAV-04    CTTAAGAGAACGTGATATGGTTTTTACTATGCCAGATAAAGAATCTGAACCTACCCCATA

GdelRNAV-01    CATGAATGATCTCGAAGCTGATTTTTTGAAGCGTGAGAATATATTTAATGAGGATACT
GdelRNAV-02    CATGAATGATCTCGAAGCTGATTTTTTGAAGCGTGAGAATATATTTAATGAGGATACT
GdelRNAV-03    CATGAATGATCTCGAAGCTGATTTTTTGAAGCGTGAGAATATATTTAATGAGGATACT
GdelRNAV-04    CATGAATGATCTCGAAGCTGATTTTTTGAAGCGTGAGAATATATTTAATGAGGATACT
```

Figure S1. Nucleotide alignment of the partial sequences of the RNA-dependent RNA polymerase of GdelRNAV viruses. Differences between sequences are enclosed in a red frame. RdRp sequences were aligned using MAFFT on Geneious 9.1.3.
